# YY1-MEDIATED PROMOTER-SUPER ENHANCER INTERACTIONS REGULATE MSC MULTIPOTENCY DURING REPLICATIVE AGING

**DOI:** 10.1093/geroni/igz038.3328

**Published:** 2019-11-08

**Authors:** Luyang Sun, Brenna McCauley, Weiwei Dang

**Affiliations:** Baylor College of Medicine, Houston, Texas, United States

## Abstract

Mesenchymal stem cells (MSC) are versatile in stem cell therapy and regenerative medicine, due to its capacity of in-vitro expansion and multilineage differentiation. However, how the MSC’s functions and the underlying epigenomic features are affected by aging and cellular senescence remains poorly understood. Here we used culture expanded human MSC isolated from umbilical cord tissue as an ex vivo model to characterize the chromatin 3D conformational changes associated with aging and senescence and correlate these changes to the changes in MSC stem cell functions during aging. By comparing late passage (P15) vs early passage (P5) MSC, no fundamental changes were found on the large-scale chromatin spatial structures including compartment switching and TAD boundaries. However, when focused on super enhancers, which act as major cis-regulatory hubs associated with lineage-specific functions, a large number of significant promoter-super enhancer interaction changes were discovered. Further examination revealed that YY1, a key regulator of promoter-enhancer looping, was highly enriched in these promoters and their corresponding super-enhancers. The expression of the genes involved in such changes is positively correlated with the variation of promoter-super enhancer interaction, indicating that the spatial communications between promoter and super-enhancer positively regulate gene expression. GO-term and TFBS enrichment analysis showed that these genes involved in cell cycle, adipocyte and osteoblast differentiation. Collectively, these results suggest that YY1-mediated promoter-super enhancer looping is an important regulatory mechanism for MSC multipotency during replicative aging.

